# Genome-Wide Identification of the PLATZ Transcription Factor Family in *Populus euphratica* Oliv. and Functional Characterisation of *PePLATZ8* in Drought Tolerance

**DOI:** 10.3390/plants15071065

**Published:** 2026-03-31

**Authors:** Tiantian Ran, Jianhao Sun, Chen Qiu, Xiaoli Han, Lijun Gao, Zhijun Li

**Affiliations:** 1 Xinjiang Production and Construction Corps Key Laboratory of Protection and Utilization of Biological Resources in Tarim Basin, College of Life Science & Technology, Tarim University, Alar 843300, China; r13688569153@163.com (T.R.); sunjianhaotea@163.com (J.S.); qiuchentea@163.com (C.Q.); lilyan0509@163.com (X.H.); 2 College of Information Engineering, Tarim University, Alar 843300, China; 10757232291@stumail.taru.edu.cn

**Keywords:** *Populus euphratica*, PLATZ transcription factor, abiotic stress, molecular breeding

## Abstract

Plant AT-rich sequence and zinc-binding (PLATZ) proteins are a class of plant-specific transcription factors that have been identified and functionally characterised in multiple species. However, the *PLATZ* gene family has not yet been systematically characterised in *Populus euphratica*. In this study, 19 *PePLATZ* genes were identified and classified into five subgroups. The analyses of gene structure, conserved motifs and protein domains indicated that the PLATZ family is highly conserved during evolution. Meanwhile, promoter *cis*-acting element profiling suggested their potential roles in stress-responsive transcriptional regulation. Transcriptomic and qRT-PCR analyses showed that *PePLATZ1*, *PePLATZ6*, *PePLATZ8*, *PePLATZ14* and *PePLATZ19* were highly expressed in the roots, stems and leaves of *P. euphratica* and were strongly induced by drought stress. *PePLATZ8* localised to the nucleus and lacked transactivation activity but acted as a transcriptional repressor in planta. Transgenic poplar lines overexpressing *PePLATZ8* exhibited significantly enhanced drought tolerance. Furthermore, after drought treatment, *PePLATZ8*-overexpressing plants accumulated high levels of H_2_O_2_ and exhibited significantly increased total superoxide dismutase activity, which likely contributed to improved drought tolerance. Together, the above findings deepen our understanding of the structure–function relationships of PePLATZ proteins and identify *PePLATZ8* as a promising candidate gene for the molecular breeding of drought-resistant poplar germplasm.

## 1. Introduction

Drought is a key abiotic stress factor affecting plant growth and development. It is a major environmental constraint on agricultural productivity and sustainable forest ecosystem development [1,2,3]. With the acceleration of global climate change, water scarcity has become a major challenge for plant survival and productivity. Under drought stress, plants activate adaptive mechanisms at multiple levels, including morphological adjustments, physiological and biochemical alterations and transcriptional or posttranscriptional regulation. Together, these coordinated responses constitute an integrated system of drought tolerance [4,5,6,7]. Under severe stress, plants build complex molecular regulatory networks. In these networks, transcriptional control by transcription factors (TFs) serves as a central regulatory layer [8,9,10]. TFs play a central role in plant stress signal transduction by modulating the expression of downstream target genes [11,12,13,14,15]. Amongst the many TF families, zinc finger proteins constitute one of the largest and serve as key regulators of plant responses to environmental stresses, particularly drought [16,17,18]. Notably, within this family, plant AT-rich sequence and zinc-binding (PLATZ) proteins represent a distinct class of plant-specific zinc finger proteins. They contain two highly conserved zinc finger motifs (C–x_2_–H–x_11_–C–x_2_–C–x_[4–5]_–C–x_2_–C–x_[3–7]_–H–x_2_–H and C–x_2_–C–x_[10–11]_–C–x_3_–C) that are necessary for DNA binding [19]. PLATZ TFs were first isolated in pea, and PsPLATZ1 binds nonspecifically to A/T-rich sequences and sup-presses the expression of target genes, acting as a transcriptional repressor [19].

PLATZ proteins are involved in tissue growth, development, seed formation, yield and responses to salt and drought. In maize, *ZmPLATZ12* is closely linked to seed development and yield [20,21]. In *Arabidopsis*, *PLATZ1* and *PLATZ4* promote drought tolerance [22,23], whereas *PLATZ2* and *PLATZ7* reduce salt stress adaptation [24]. *GhPLATZ1* affects seed germination in upland cotton and the responses of transgenic *Arabidopsis* seedlings to salt and osmotic stress [25]. Similarly, in soybean, *GmPLATZ17* reduces drought tolerance by modulating the regulatory pathway of the stress-responsive TF *GmDREB5* [26]. Collectively, the above studies demonstrate that PLATZ TFs are key regulators of plant growth, development and abiotic stress responses. However, the functional characterisation of PLATZ TFs in woody plants remains largely unexplored. This knowledge gap is particularly evident in species adapted to the arid and desert ecosystems of Northwest China, where investigations on the *PLATZ* gene family are scarce and the underlying molecular mechanisms of stress adaptation have not yet been systematically elucidated.

*Populus euphratica* Oliv., a member of the genus Populus (*Salicaceae*), is predominantly distributed in the arid regions of Xinjiang, Gansu and Inner Mongolia, where it serves as a dominant pioneer species in desert ecosystems [27]. It has become an important model for investigating stress-resistance mechanisms in woody plants because of its exceptional tolerance to drought and salinity [28,29,30,31,32]. However, under natural conditions, prolonged drought still severely restricts its growth, development and regeneration capacity [33,34]. Therefore, identifying drought-responsive genes is essential for elucidating the molecular basis of stress adaptation and provides a foundation for developing stress-resilient *P. euphratica* through molecular breeding.

Although PLATZ proteins have been shown to participate in stress responses in various plants, the *PePLATZ* gene family has yet to be systematically identified and functionally elucidated, and its biological role remains to be further clarified. On the basis of the whole-genome data of *P. euphratica*, this study identified 19 *PePLATZ* family members and systematically analysed their phylogenetic relationships, gene structures, conserved domains and motif compositions, promoter *cis*-acting elements, subcellular localisations and transcriptional activities. Furthermore, five *PePLATZ* genes were selected for the analysis of their tissue expression patterns under drought stress. In combination with transcriptome analysis, this study heterologously overexpressed the candidate gene *PePLATZ8* in *Populus deltoides* × *Populus euramericana* cv. ‘Nanlin895’ and preliminarily validated its function in drought response. Unlike previous studies focusing mainly on *PLATZ* gene identification in model species, this study integrates gene family analysis with functional verification of *PePLATZ8* under drought stress in *Populus euphratica*, providing new insights into the regulatory mechanisms of *PLATZ* genes in desert-adapted woody plants. In summary, this study can not only provide basic data to elucidate the structural characteristics of the *PePLATZ* gene family in *P. euphratica* and its potential biological functions in stress response but can also provide candidate gene resources for the molecular improvement of drought tolerance and breeding of stress-resistant *Populus* varieties.

## 2. Results

### 2.1. Genome-Wide Identification of PLATZ Genes in P. euphratica

A BLASTP search was performed against the *P. euphratica* genome by using Arabidopsis PLATZ protein sequences as queries. Following this search, candidate sequences were further validated on the basis of the presence of conserved PLATZ domains identified by using the PFAM and SMART databases. As a result, 19 *PePLATZ* genes were identified and named as *PePLATZ1*–*PePLATZ19* in accordance with their chromosomal positions. Chromosomal localization analysis revealed that the 19 *PePLATZ* genes were unevenly distributed across 12 chromosomes of *Populus euphratica* (Figure S1). Chromosome 1 harbored the highest number of *PePLATZ* genes (three members), while chromosomes 3, 5, 6, 14, and 18 each contained two genes. The remaining *PePLATZ* genes were individually located on chromosomes 2, 8, 9, 10, 13, and 19. This distribution pattern indicates that the *PePLATZ* gene family is relatively dispersed throughout the *P. euphratica* genome. In addition, no obvious tandemly duplicated gene pairs were identified among the *PePLATZ* genes, suggesting that tandem duplication was not the primary driving force underlying the expansion of this gene family.

Subsequent physicochemical analyses showed that the PePLATZ proteins ranged from 173 to 317 amino acids in length and had predicted molecular weights of 19.9–37.0 kDa. Among them, PePLATZ1 encodes the smallest protein (173 amino acids), whereas PePLATZ13 represents the largest member (317 amino acids). The predicted isoelectric points ranged from 7.1 to 9.4, indicating that most PePLATZ proteins are basic. In addition, all PePLATZ proteins were predicted to be unstable (instability index > 40) and mostly hydrophilic (GRAVY: −0.947 to −0.3), with aliphatic indices ranging from 59.1 to 101. Subcellular localisation prediction further indicated that most PePLATZ proteins are localised in the nucleus, supporting their potential roles as transcriptional regulators (Table S1).

### 2.2. PLATZ Protein Cluster Analysis

**Figure 1:**
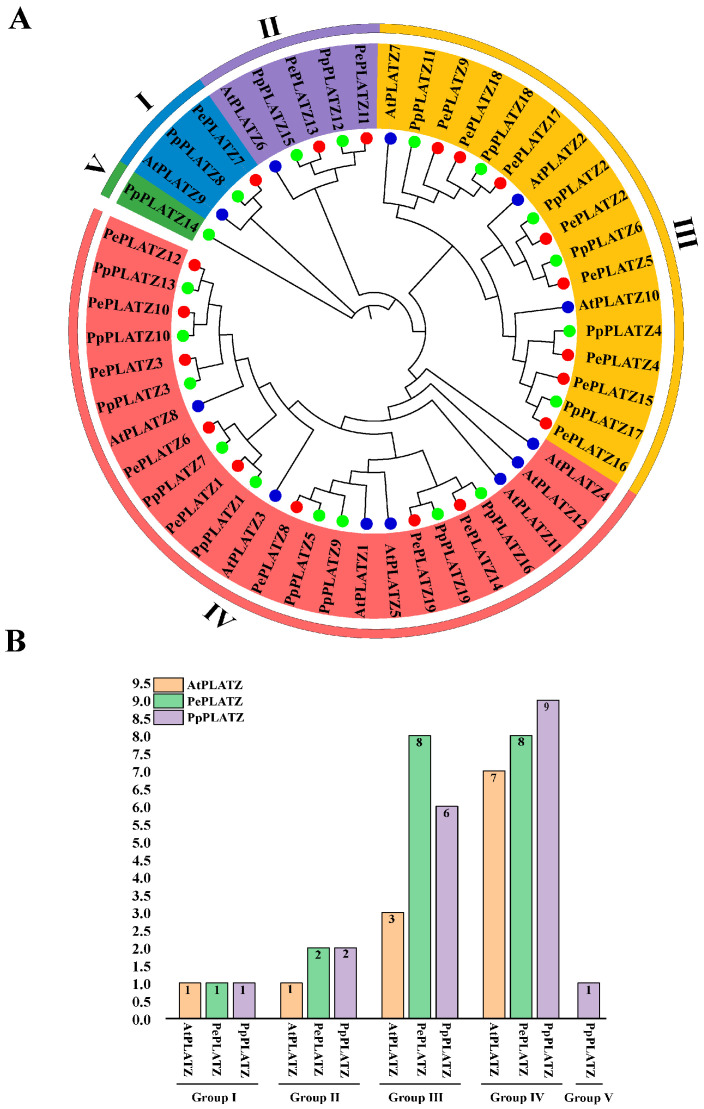
Phylogenetic analysis and classification of PLATZ gene family members. (**A**) Phylogenetic tree of PLATZ proteins from different species. The proteins are divided into five groups (Group I–V). Different colored dots represent different species: AtPLATZ (*A. thaliana*), PePLATZ (*P. euphratica*), and PpPLATZ (*P. pruinosa*). (**B**) Distribution of PLATZ genes in different groups. The bar chart shows the number of PLATZ genes from each species in Groups I–V. Different colors represent different species.

Amino acid sequences from 19 PePLATZ, 19 PpPLATZ and 12 AtPLATZ proteins were aligned to explore the phylogenetic evolutionary relationships between PePLATZ genes and their homologues in other species, and a phylogenetic tree was constructed (Figure 1A). The results showed that the 50 PLATZ proteins were divided into five subgroups (groups I–V) (Figure 1B). The *PLATZ* genes of *P. euphratica* showed high sequence similarity with those of other Salicaceae species, particularly *Populus pruinosa*, indicating the strong conservation of PLATZ proteins throughout evolution. Notably, AtPLATZ1 and PePLATZ8 were identified as homologous proteins (Figure 1A), suggesting that they may perform similar biological functions.

### 2.3. Gene Structure, Sequence Motifs and Conserved Domain Analysis

**Figure 2:**
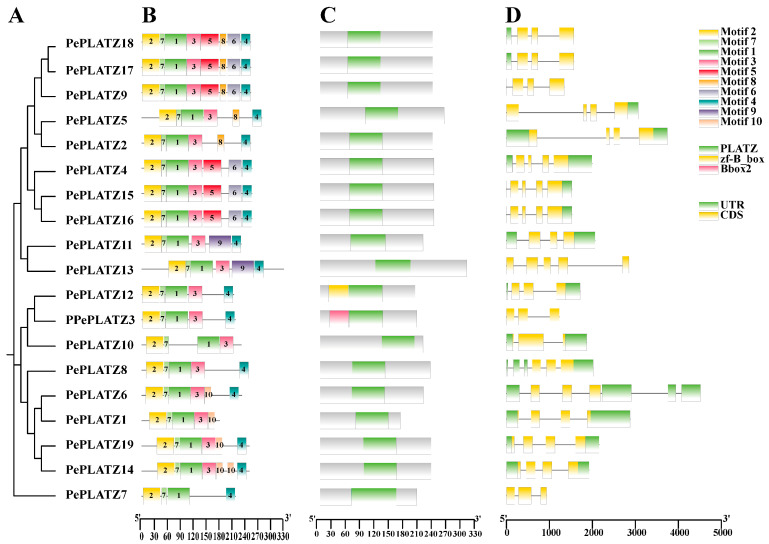
Sequence motif, conserved domain and gene structure analyses of PLATZ families in *P. euphratica*. (**A**) Phylogenetic tree of 19 PLATZ protein sequences constructed by using TBtools software (version 2.119). (**B**) Motif composition of PLATZ proteins. The 10 conserved motifs in PLATZ proteins are shown in differently coloured boxes numbered 1–10. (**C**) Conserved domain composition of PLATZ proteins. (**D**) Exon/intron structure of *PLATZ* genes.

A systematic analysis of gene structures, conserved motifs and domain architectures was performed to clarify the conserved features and potential functional divergence of the *PLATZ* family in *P. euphratica* (Figure 2). Ten conserved motifs (motifs 1–10) were identified (Figure 2B) on the basis of multiple sequence alignments. These motifs were displayed graphically on the basis of their phylogenetic relationships. The number and organisation of motifs varied considerably amongst subgroups, with individual proteins containing 4–8 motifs. Amongst these motifs, 1, 2, 3 and 7 were highly conserved in most members. Motif 4 was present in the majority of proteins and typically located near the C-terminus of the PLATZ domain. This characteristic suggests that motif 4 might serve as an indicator of domain integrity. *PePLATZ3* and *PePLATZ12* contained additional Bbox2 and zf-B_box domains (Figure 2C), respectively, in addition to the typical PLATZ domain. The further analysis of gene structure revealed that the number of exons ranged from three to five across 19 members. Four genes lacked UTR regions (Figure 2D). These results may reflect intron/exon gain or loss events in PLATZs during evolution. The conserved and divergent features amongst different subgroups likely reflect the functional diversification of PePLATZ proteins in *P. euphratica.*

### 2.4. Promoter Cis-Acting Element Analysis

**Figure 3:**
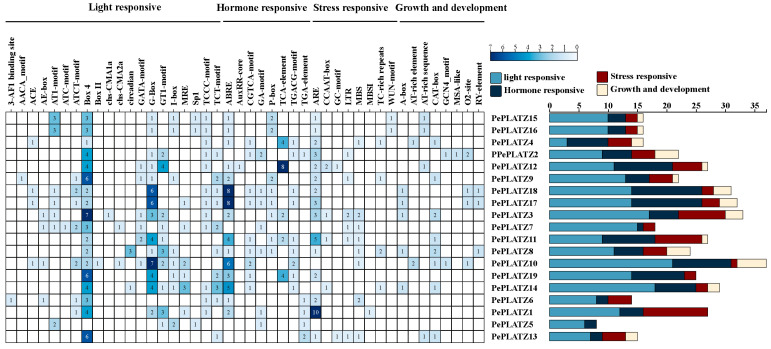
Analysis of *cis*-acting elements in the upstream promoter regions of *PePLATZ* genes. The number represents the number of *cis*-acting elements. Different categories are shown with different colour blocks.

The promoter regions of *PePLATZ* genes were analysed by using the PlantCARE database to identify *cis*-acting regulatory elements. This analysis helped infer their potential transcriptional regulatory features and expression control mechanisms (Figure 3). The identified *cis*-acting elements fell into the following categories: stress-responsive elements (e.g., defence, low temperature, wound and drought response elements), hormone-responsive elements (e.g., gibberellin [GA], auxin, methyl jasmonate [MeJA] and salicylic acid response elements), light-responsive elements and growth-related elements (e.g., endosperm and meristem response elements). Abscisic acid (ABA)–responsive elements (ABREs), CGTCA and TGACG motifs (related to jasmonic acid response), anaerobic inducible elements (AREs) and MBS (drought-induced response elements) were highly represented in PePLATZ promoter regions. Together, these results suggest that *PePLATZ* genes might play potential roles in hormone and abiotic stress responses.

### 2.5. Synteny Analysis of the PePLATZ Gene Family

**Figure 4:**
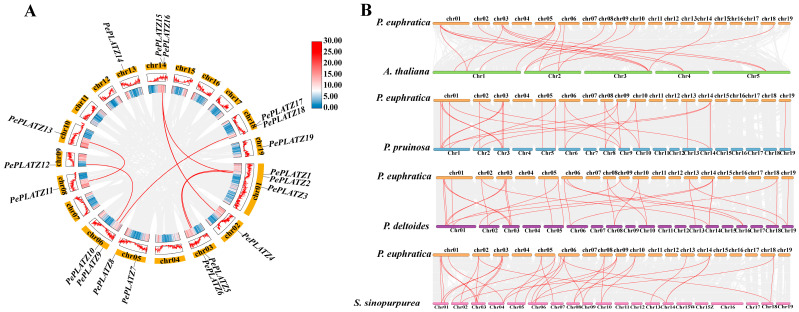
Collinearity analysis of multispecies *PLATZ* genes. (**A**) Intraspecific covariance analysis of *PePLATZ* genes. (**B**) Collinearity analysis of *PLATZ* genes between *P. euphratica* and five other species (*P. deltoides*, *P. pruinosa*, *S. sinopurpurea* and *A. thaliana*). Grey lines in the background indicate the collinear blocks within *P. euphratica* and other plant genomes, and red lines highlight collinear *PLATZ* pairs.

Intra- and interspecific collinearity analyses were conducted to resolve the evolutionary forces acting on the *PePLATZ* family (Figure 4). Intraspecific analysis identified seven segmentally duplicated gene pairs: *PePLATZ1/PePLATZ6*, *PePLATZ4/PePLATZ15*, *PePLATZ2/PePLATZ15*, *PePLATZ10/PePLATZ12*, *PePLATZ2/PePLATZ5*, *PePLATZ9/PePLATZ17* and *PePLATZ11/PePLATZ13*. These 14 genes lacked tandem organisation (Figure 4A), indicating that expansion occurred through large-scale duplication rather than local amplification. *PePLATZ8* remained a single copy, suggesting a distinct evolutionary trajectory. Interspecific synteny between *P. euphratica* and *Arabidopsis thaliana*, *P. pruinosa*, *Populus deltoides* and *Salix sinopurpurea* revealed 19, 30, 32 and 34 collinear *PePLATZ* gene pairs, respectively (Figure 4B). These findings are consistent with the stronger conservation of *PLATZ* loci within *Populus* and other Salicaceae members than within *A. thaliana.*

### 2.6. Analysis of Natural Selection Pressure and Protein Structure of PePLATZs

**Figure 5:**
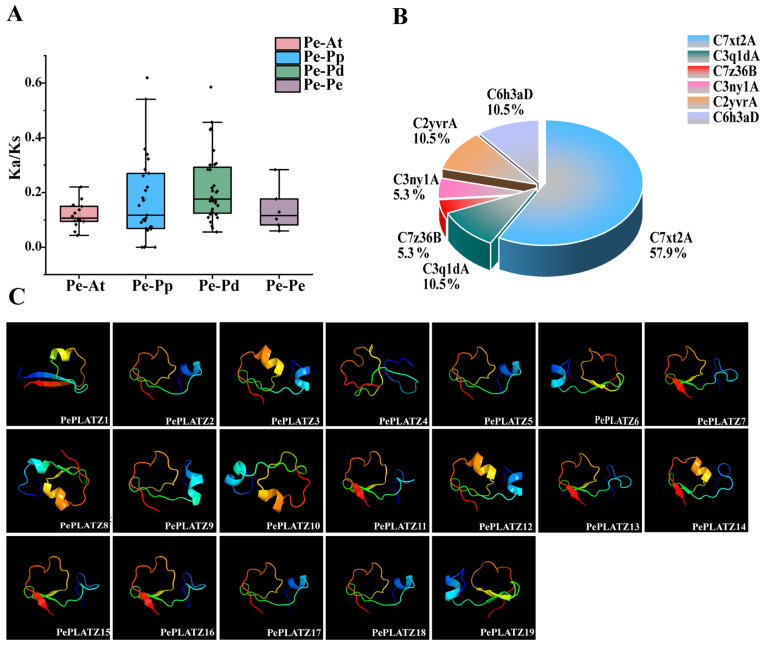
Analysis of protein evolutionary rates and three-dimensional structures. (**A**) Ka/Ks ratios of homologous gene pairs in *P. euphratica*, *A. thaliana*, *P. pruinosa* and *P. deltoides*. The dots represent individual gene pairs. (**B**) Number of protein models. (**C**) Predicted three-dimensional structures of PePLATZ proteins (confidence value > 85%). The colors represent the protein structure from the N-terminus (blue) to the C-terminus (red).

This study compared the orthologous gene relationships among *P. euphratica*, *A. thaliana*, *P. pruinosa*, and *P. deltoides* to investigate the evolutionary patterns of the *PLATZ* gene family in *P. euphratica* (Figure 5A). A total of 88 orthologous gene pairs were identified, and all Ka/Ks ratios were less than 1 (Table S2), indicating that these genes have predominantly undergone purifying selection during evolution. In addition to the orthologous gene analysis, seven paralogous gene pairs were identified within *P. euphratica* (Table 1). Except for the *PePLATZ2/PePLATZ15* pair, which may have experienced synonymous substitutions at certain loci, the Ka/Ks ratios of the remaining six paralogous pairs ranged from 0.05 to 0.2. These results further suggest that the *PLATZ* gene family in *P. euphratica* has been subjected to strong purifying selection during its evolutionary history.

To further explore the evolutionary dynamics of these duplicated genes, the divergence times of the paralogous gene pairs were estimated based on Ks values (Table 1). The estimated divergence times ranged from approximately 88,500 to 637,000 years ago, suggesting that these duplication events likely occurred relatively recently in the evolutionary history of Populus, rather than being associated with ancient whole-genome duplication events.

Shifting from evolutionary timing to structural analysis, the tertiary structures of PePLATZ proteins were predicted using the Phyre2.2 server (confidence > 85%) (Figure 5C). The results showed that all PePLATZ proteins contain zinc finger domains. The predicted secondary structural components included α-helices, β-turns, extended strands and random coils. Six structural types of PePLATZ proteins were predicted, including C7xt2A, C3q1dA, C7z36B, C3ny1A, C2yvrA, and C6h3aD, among which C7xt2A was the most common type (Figure 5B). Some PePLATZ proteins, such as PePLATZ15 and PePLATZ16 as well as PePLATZ17 and PePLATZ18, exhibited highly similar predicted three-dimensional structures, suggesting a conserved structural pattern within the PLATZ protein family.

### 2.7. Expression Pattern of PePLATZ Under Drought Stress

**Figure 6:**
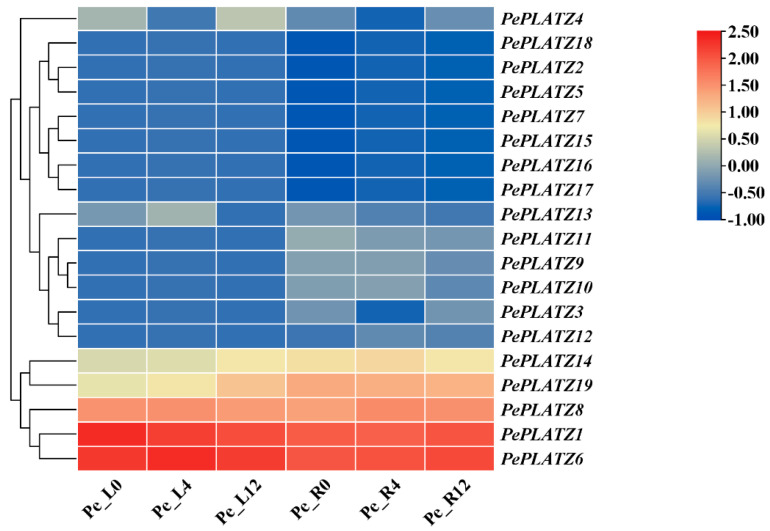
Expression patterns of *PePLATZ* genes. Transcriptome analysis of *PePLATZ* gene expression under drought stress at 0, 4 and 12 h. The colour scale represents relative gene expression levels, with red indicating high expression and blue indicating low expression.

The transcriptome profiles of leaf and root tissues were analysed at 0, 4 and 12 h following drought treatment to elucidate the responses of *PePLATZ* genes to drought stress (Figure 6). *PePLATZ6*, *PePLATZ8*, *PePLATZ14* and *PePLATZ19* were consistently upregulated in roots and leaves over the 0–12 h period, whereas *PePLATZ1* displayed root-specific induction under drought. The remaining family members were either downregulated or transcriptionally silent under these conditions.

### 2.8. Expression Patterns of PePLATZ Genes Under Drought Stress

**Figure 7:**
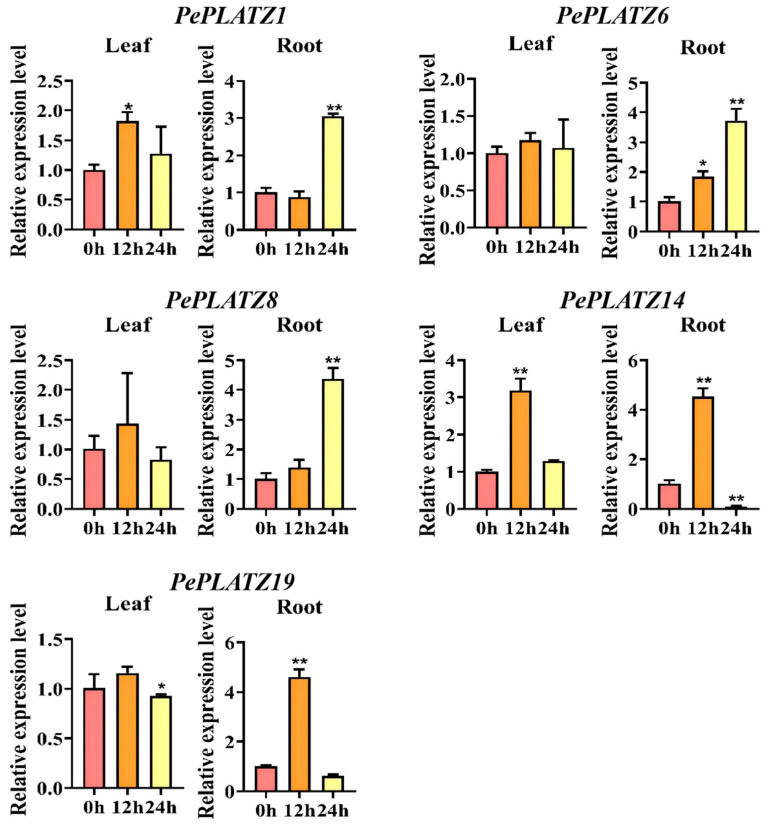
qRT-PCR analysis of *PePLATZ* under PEG6000 treatment. Error lines represent the mean ± standard error of the three biological replicates for qRT-PCR analysis. Different letters indicate significant differences between groups. * *p* < 0.05; ** *p* < 0.01.

Under the guidance of transcriptome analysis, five *PePLATZ* genes, namely, *PePLATZ1*, *PePLATZ6*, *PePLATZ8*, *PePLATZ14* and *PePLATZ19*, that were highly expressed across tissues and responsive to drought were selected for validation. Their expression levels in roots and leaves under control and drought treatments were quantified by using quantitative real-time PCR (qRT-PCR) (Figure 7). Under drought conditions, all five genes exhibited tissue-specific dynamics. In leaves, the expression of all *PePLATZ* genes was significantly elevated at 12 h under drought treatment relative to under the control treatment. In roots, *PePLATZ1*, *PePLATZ6* and *PePLATZ8* peaked at 24 h, whereas *PePLATZ14* and *PePLATZ19* showed maximal induction at 12 h. Collectively, these patterns indicate the coordinated, tissue-dependent response of *PePLATZ* family members to drought stress. Such a response is consistent with functional redundancy and/or conserved roles in mediating drought adaptation.

### 2.9. Protein Interaction Network Analysis

Protein–protein interaction (PPI) network analysis was conducted on PePLATZ8 by using STRING (Figure S2). PePLATZ8 exhibited high sequence similarity to the A. thaliana protein AtPLATZ1. The predicted interaction network comprised multiple TFs, such as zinc-finger proteins (COL10, COL15 and DOF1.3), homeobox-leucine zipper proteins (ATHB-21), NAC domain–containing factors (NAC041 and NAC0074), B3 domain–containing proteins (F18O9.70, MGO3.2 and REM22) and auxin response factors (ARF31). These results imply that PePLATZ8 may regulate plant growth, development and stress responses through interactions with these TFs.

### 2.10. Analysis of PePLATZ8 Promoter Activity

The prediction of promoter *cis*-acting elements revealed that the *PePLATZ8* promoter region contains two GA-responsive elements, two MeJA-responsive elements and one ABA-responsive element (ABRE) (Figure S3A). These hormone-related *cis*-elements are commonly associated with stress-responsive transcriptional regulation. Consistent with this prediction, RNA-seq and qRT-PCR analyses showed that *PePLATZ8* expression was significantly induced under drought conditions (Figure 7). To further investigate the regulatory activity of the *PePLATZ8* promoter, promoter–GUS assays were performed. GUS staining revealed that GUS activity in tobacco leaves was markedly enhanced after seven days of drought treatment compared with the control (Figure S3B). Among the hormone treatments, ABA induced the strongest transcriptional activation, followed by GA_3_, whereas MeJA induced a relatively weaker response. These expression patterns are consistent with the presence of ABRE and other hormone-responsive elements in the promoter region. Similar promoter architectures containing ABA- and drought-responsive *cis*-elements have also been reported in *MdPLATZ* genes, which exhibit strong transcriptional induction under water deficit conditions. Taken together, these results suggest that the *PePLATZ8* promoter is responsive to drought and multiple hormone signals, particularly ABA, supporting the hypothesis that *PePLATZ8* participates in drought stress responses in *P*. *euphratica*.

### 2.11. Subcellular Localisation of PePLATZ8

**Figure 8:**
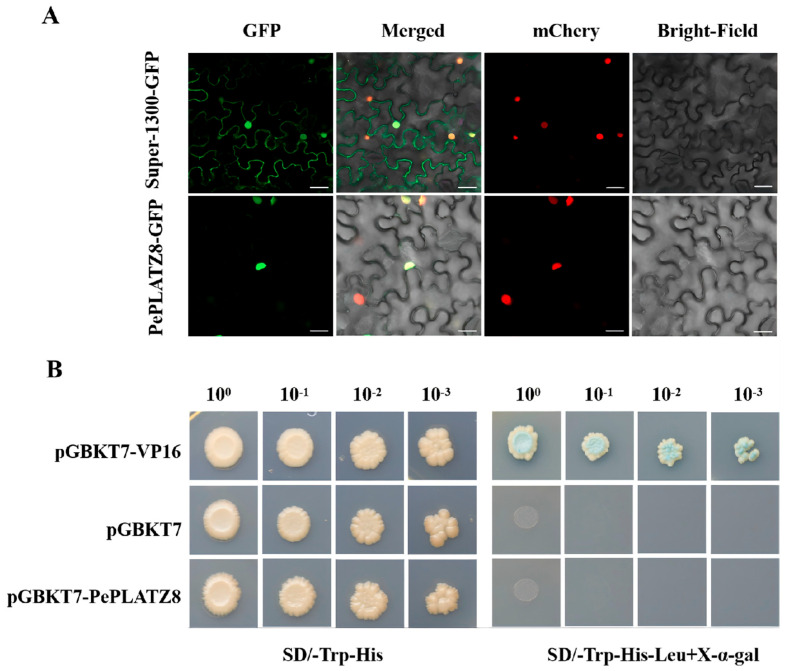
Subcellular localisation and transcriptional activity of PePLATZ8. (**A**) Subcellular localisation of the PePLATZ8–GFP fusion protein transiently expressed in tobacco leaf epidermal cells. Images show GFP fluorescence, bright-field and merged views of the GFP control and PePLATZ8–GFP. Scale bar = 20 μm. (**B**) Transcriptional activity assay of PePLATZ8 in yeast. The pGBKT7 empty vector and pGBKT7–VP16 were used as the negative and positive controls, respectively.

Studying the subcellular localisation of proteins helps determine their cellular location and infer their possible functions [35]. According to subcellular localisation prediction by WoLF-PSORT, the PePLATZ8 protein localises to the nucleus, a finding that aligns with previously reported results [1,36]. The *PePLATZ8* coding sequence was coexpressed with the empty vector pCAMBIA1300 Super:GFP in tobacco cells to verify the above prediction. Confocal laser microscopy revealed that the empty vector emitted green fluorescence throughout tobacco cells, indicating that GFP expression was normal. By contrast, the green fluorescence emitted by the PePLATZ8–GFP fusion protein was limited to the nucleus (Figure 8A). These results confirm that the PePLATZ8–GFP fusion protein specifically localises to the nucleus, supporting the possibility that PePLATZ8 functions as a TF within the nucleus.

### 2.12. Transcriptional Activity Analysis

A fusion expression vector containing the PePLATZ8 protein was transformed into Y2HGold yeast cells to elucidate whether PePLATZ8 possesses transcriptional activity. The results showed that experimental yeast cells (harbouring pGBKT7–PePLATZ8) and negative control cells (harbouring pGBKT7) grew well on SD/-Trp-His-deficient medium (Figure 8B). However, on SD/-Trp-His-Leu+X-α-gal medium, only the positive control (yeast cells carrying a vector known to induce autoactivation) exhibited normal growth and produced blue colouration, whereas neither the experimental nor the negative control cells could grow normally. These results indicate that PePLATZ8 lacks transcriptional autoactivation activity in yeast.

### 2.13. Overexpression of PePLATZ8 Enhances Plant Tolerance to Osmotic Stress

**Figure 9:**
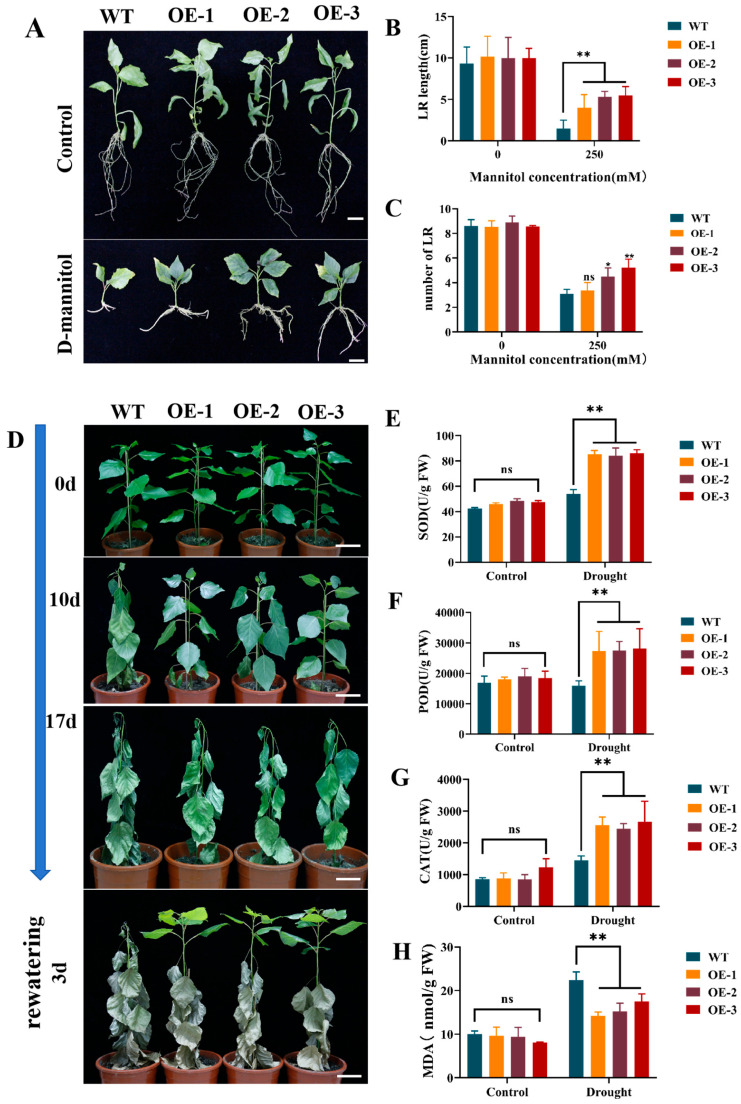
*PePLATZ8* overexpression enhances drought tolerance in plants. (**A**) Phenotypes of WT and transgenic overexpression lines (OE-1, OE-2 and OE-3) under normal and osmotic stress conditions (250 mM D-mannitol). Scale bar = 1 cm. (**B**) Statistical analysis of the initial root length of WT and OE lines under 0 and 250 mM mannitol treatments. (**C**) Statistical analysis of the lateral root number of WT and OE lines under 0 and 250 mM mannitol treatments. (**D**) Phenotypic dynamics of WT and *PePLATZ8*-OE lines during 0, 10 and 17 days of drought stress and three days after rehydration. Scale bar = 5 cm. (**E**–**H**) Assays of SOD, POD, MDA and CAT activities. Data are presented as mean ± SD from three independent biological replicates, with at least three plants per genotype included in each replicate (*n* = 3). Statistical significance: ns, not significant; * *p* < 0.05; ** *p* < 0.01.

Wild-type (WT) plants were compared with lines overexpressing *PePLATZ8* (*PePLATZ8*-OE) under mannitol-induced stress to evaluate the function of *PePLATZ8* in osmotic stress responses. Osmotic stress was simulated by adding 250 mM mannitol to the rooting medium. Tissue culture seedlings of WT and *PePLATZ8*-OE plants with comparable sizes and vigour were selected to ensure uniform initial growth, and apical shoots of equal height were subcultured. Root initiation was observed after approximately 10 days, and distinct morphological differences became apparent by 20 days (Figure 9A). WT plants developed short primary roots with an average length of 3 cm, whereas *PePLATZ8*-OE plants developed longer primary roots (5 cm) and produced more lateral or fibrous roots than WT plants (Figure 9B,C). Together, these results indicate that *PePLATZ8* overexpression enhances root development under mannitol-induced osmotic stress.

### 2.14. Overexpression of PePLATZ8 Confers Enhanced Drought Tolerance in Poplar

Transgenic *PePLATZ8*-OE and WT poplar plants were subjected to drought stress to investigate the role of *PePLATZ8* in drought tolerance. Prior to stress, both plant types exhibited comparable growth and morphology. After 10 days of drought, WT plants began to wilt, whereas transgenic plants maintained green leaves and normal physiological status. By Day 17, WT plants displayed severe dehydration, including terminal bud wilting and extensive leaf desiccation, whereas *PePLATZ8*-OE plants showed only mild wilting, with viable terminal buds and intact stems. Upon rehydration, WT leaves exhibited severe yellowing and vitality loss, whereas transgenic plants retained viable terminal buds and showed minimal leaf damage (Figure 9D). Physiological analyses indicated that drought-induced oxidative damage was mitigated in transgenic plants. The content of malondialdehyde (MDA) was significantly reduced (Figure 9H), whereas the activities of antioxidant enzymes, including peroxidase (POD), superoxide dismutase (SOD) and catalase (CAT), were markedly enhanced in *PePLATZ8*-OE plants compared with those in the WT (Figure 9E–G).

**Figure 10:**
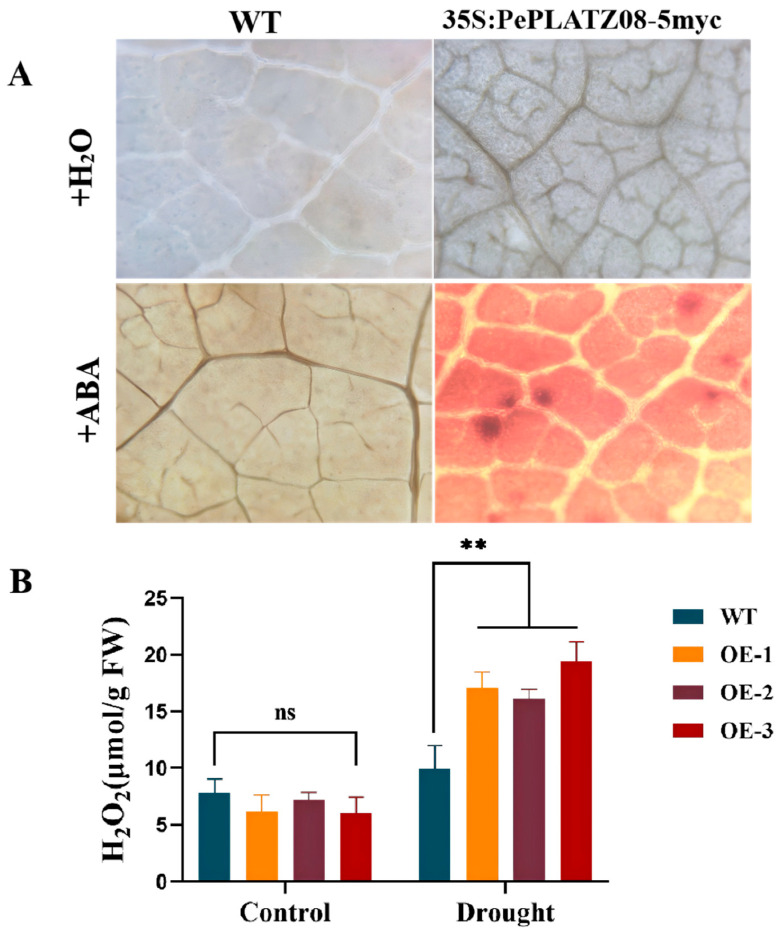
Changes in H_2_O_2_ levels under ABA treatment. (**A**) DAB staining of WT and *PePLATZ8*-overexpressing plants after ABA treatment. (**B**) Quantification of H_2_O_2_ content under ABA treatment. Data are presented as mean ± SD from three independent biological replicates, with at least three plants per genotype included in each replicate. Statistical significance was determined using one-way ANOVA followed by Tukey’s multiple comparison test (ns, not significant; ** *p* < 0.01).

H_2_O_2_ can be detected in plant tissues by using 3,3-diaminobenzidine (DAB) through the generation of brown compounds under the catalysis of peroxidase. *PePLATZ8*-OE and WT plants were treated with ABA or dehydration, and then their leaves were stained with DAB. DAB staining revealed that under normal watering conditions, the leaves of WT and *PePLATZ8*-OE plants were pale overall without notable brown precipitates. Only slightly intensified signals were detected around specific leaf cells in overexpressing plants. By contrast, after ABA treatment, pronounced brown precipitates were observed in the leaves of both plant types (Figure 10A). However, the overexpressing plants had markedly higher staining intensity than the WT plants, indicating that their H_2_O_2_ levels were elevated (Figure 10B). DAB staining and quantitative H_2_O_2_ measurements further revealed that drought treatment enhanced H_2_O_2_ accumulation, with transgenic plants displaying stronger signals than WT plants. Collectively, these results suggest that *PePLATZ8* overexpression enhances drought tolerance by alleviating membrane lipid peroxidation, boosting antioxidant defences and modulating H_2_O_2_-mediated stress responses.

## 3. Discussion

*P. euphratica*, a dominant tree species in desert oases and a typical relict lineage of arid environments, has emerged as a model system for investigating the mechanisms underlying abiotic stress tolerance in woody plants owing to its remarkable resistance to drought and salinity [37]. Nevertheless, water deficit remains a major ecological constraint that limits seedling establishment and impedes the regeneration of natural populations of this species. PLATZ proteins are a class of zinc-dependent DNA-binding TFs considered plant-specific regulators of gene expression [38,39]. Accumulating evidence indicates that PLATZ TFs play dual roles in plant growth and development [39,40,41] and adaptive responses to drought stress [22,24,25]. However, the genomic composition, structural characteristics and functional roles of the *PLATZ* gene family in *P. euphratica* have not yet been systematically characterised. Compared with previously reported *PLATZ* members, *PePLATZ8* appears to play a conserved yet potentially species-specific role in regulating drought tolerance in *Populus* species. Therefore, this study identified 19 *PLATZ* family members in the *P. euphratica* genome and further characterised the functional properties of a representative gene, *PePLATZ8*. Notably, this work demonstrates that the overexpression of *PePLATZ8* enhances drought tolerance in transgenic poplar, at least in part by limiting cell membrane damage and promoting antioxidant enzyme activities.

A phylogenetic tree constructed using PLATZ protein sequences from *P. euphratica*, *P. pruinosa*, *Populus trichocarpa* and *A. thaliana* clarified the evolutionary relationships among PLATZ proteins (Figure 1A). The 50 PLATZ members identified were classified into five groups (Figure 1B). The group distribution suggests close evolutionary relationships among PLATZ proteins. Notably, AtPLATZ1 and PePLATZ8 were identified as homologous proteins (Figure 1A). Previous studies have shown that AtPLATZ1 positively regulates drought tolerance in *Arabidopsis*; its overexpression enhances plant survival under water deficit, whereas platz1 mutants display reduced tolerance [22,25,42]. These observations suggest that PePLATZ8 may share partially conserved roles with AtPLATZ1 in drought stress responses. Further analysis of conserved motifs, domains and gene structures revealed that *PePLATZ* genes within the same clade share similar gene structures and conserved motifs, whereas genes in different clades exhibit substantial divergence (Figure 2). These differences indicate that *PLATZ* genes may have undergone considerable functional diversification during evolution. Intron–exon structure analysis also revealed substantial variation in intron number and length among *PLATZ* genes (Figure 2D). Introns are known to contribute to genome evolution by buffering coding sequences against mutations and facilitating the emergence of novel genes [43]. Most PLATZ proteins contain a single PLATZ domain, whereas only a minority possess additional BBOX domains (Figure 2C), a structural feature consistent with PLATZ families reported in other plant species [1,42]. The integration of additional domains may therefore contribute to the diversification and functional expansion of PLATZ proteins. Consistent with previous findings [44], these results suggest that *PLATZ* genes share a common ancestry, and evolutionary rate analysis further indicates that they have been subjected to strong purifying selection (Table 1).

Promoter regions of many stress-responsive genes contain diverse *cis*-acting regulatory elements involved in environmental adaptation [45,46,47]. In the present study, the promoters of several *PePLATZ* genes were predicted to harbour multiple stress-responsive and hormone-related *cis*-elements (Figure 3), supporting their potential involvement in abiotic stress responses. For instance, numerous ABREs were detected, suggesting that *PePLATZ* genes may participate in ABA-mediated signalling pathways during drought stress. In addition, several low-temperature-responsive (LTR) and drought-responsive (MBS) elements were identified. These *cis*-elements may function as molecular switches that regulate gene expression under adverse environmental conditions. Importantly, the enrichment of ABRE, MeJA- and GA-related *cis*-elements in the *PePLATZ8* promoter further supports its potential involvement in hormone-mediated drought responses and may explain the stress-induced expression patterns observed in this study. This regulatory architecture is consistent with previous reports showing that *PLATZ* family members in various plant species possess similar stress-responsive promoter elements [44,48]. Collinearity analysis further revealed strong syntenic relationships between *P. euphratica* and *Salix sinopurpurea*, whereas weaker collinearity was detected with *A. thaliana* (Figure 4). The larger number of *PLATZ* genes in poplar compared with *Arabidopsis* may reflect the adaptive evolution of woody species in arid environments.

Five candidate genes (*PePLATZ1*, *PePLATZ6*, *PePLATZ8*, *PePLATZ14* and *PePLATZ19*) were selected on the basis of RNA-seq data to investigate the potential roles of *PePLATZ* genes in abiotic stress responses. qRT-PCR analysis confirmed that these genes were strongly induced under drought conditions (Figure 7), indicating that they are responsive to water deficit. Increasing evidence suggests that members of the *PLATZ* gene family play important roles in plant stress tolerance [26,41,49,50]. For example, *MdPLATZ* in *Malus domestica* contributes significantly to drought tolerance [50], whereas overexpression of *GhPLATZ1* enhances osmotic and salt stress resistance in *Arabidopsis* [25]. Similarly, several *PLATZ* genes in *Carya illinoinensis* show strong induction under drought stress [1]. Given that PLATZ proteins often function as transcriptional regulators, *PePLATZ8* may modulate the expression of downstream stress-responsive genes involved in hormone signalling or reactive oxygen species (ROS) homeostasis. This hypothesis is supported by the presence of hormone-related *cis*-elements in its promoter and its drought-induced expression pattern. However, further studies are required to identify the direct downstream targets of *PePLATZ8*.

Given that PLATZ proteins function as transcription factors, the transcriptional activity of PePLATZ8 was further examined (Figure 8A). Yeast cells harbouring the PePLATZ8–pGBKT7 fusion construct did not grow on selective SD/-Trp-His-Leu+X-α-gal medium, similar to cells containing the empty vector. This result indicates that PePLATZ8 lacks transcriptional activation activity in yeast, which is consistent with previous findings for other PLATZ proteins [1,26]. In addition, subcellular localisation analysis confirmed that PePLATZ8 is localised in the nucleus (Figure 8B), further supporting its potential role as a transcriptional regulator.

Drought stress is one of the most critical environmental factors limiting plant growth and productivity [36]. Because a stable genetic transformation system has not yet been established for *P. euphratica*, the heterologous expression of *PePLATZ8* was performed in the poplar cultivar ‘Nanlin895’, a closely related species (Figure 9). Root development plays a crucial role in plant growth, resource acquisition and stress adaptation [51,52]. Primary root elongation during early seedling development is particularly sensitive to environmental stress [53,54]. In this study, mannitol-induced osmotic stress significantly inhibited root growth in both WT and transgenic plants. However, *PePLATZ8*-OE lines consistently developed longer primary roots than WT plants under stress conditions (Figure 9A–C). Under drought treatment, transgenic plants also exhibited delayed leaf wilting and stronger recovery after rehydration (Figure 9D). Similar phenotypes have been reported for other *PLATZ* overexpression lines, such as *PhePLATZ1*-OE plants in *Arabidopsis*, which display reduced wilting and improved survival after drought stress [36]. These observations collectively indicate that *PePLATZ8* positively regulates drought tolerance in poplar.

Reactive oxygen species (ROS) production represents a key physiological response of plants to abiotic stress [55]. Excessive ROS accumulation can damage cellular structures and metabolic processes, whereas moderate ROS levels may function as signalling molecules in stress responses. Plants therefore rely on antioxidant defence systems, including enzymes such as SOD, POD and CAT, to maintain ROS homeostasis [56,57].

In this study, drought stress led to higher H_2_O_2_ accumulation in transgenic plants than in WT controls (Figure 10A,B). The activities of CAT, POD and SOD also increased significantly (Figure 9E–G). These enzyme changes helped maintain normal ROS levels and supported the normal growth and development of *PePLATZ8* plants. Notably, *PePLATZ8*-OE plants exhibited increased total SOD activity in response to elevated H_2_O_2_ after drought treatment. This response may enhance drought resistance. Moderate accumulation of H_2_O_2_ is widely recognised as an important signalling molecule in plant stress responses. In this study, although H_2_O_2_ levels increased under drought stress, the enhanced antioxidant enzyme activities in OE plants may help maintain ROS homeostasis, allowing H_2_O_2_ to function as a signalling molecule rather than causing oxidative damage. Furthermore, MDA levels were significantly lower in transgenic plants than in WT plants (Figure 9H). This finding indicates reduced oxidative damage to their cell membranes. *PePLATZ8* enhances drought tolerance by mitigating membrane damage, reducing water loss and boosting antioxidant defences.

Although the present study provides important insights into the role of *PePLATZ8* in drought tolerance, the underlying regulatory mechanisms remain to be fully elucidated. Future studies could employ CRISPR/Cas9-mediated genome editing to investigate the regulatory functions of *PePLATZ8* in greater detail. For example, targeted editing of the *PePLATZ8* promoter may help determine how specific ABA-responsive *cis*-elements contribute to drought-induced expression. In addition, identifying transcriptional partners or downstream targets of *PePLATZ8*, particularly genes involved in ROS homeostasis or ABA signalling pathways, will provide a deeper understanding of its regulatory network and facilitate the development of drought-resilient woody plants.

## 4. Materials and Methods

### 4.1. Identification of the PLATZ Gene Family

A species-specific protein database was constructed based on the complete genome sequence of *Populus euphratica*. Putative PePLATZ proteins were identified using two complementary approaches. First, protein sequences of the *Arabidopsis thaliana PLATZ* gene family were retrieved from the TAIR11 database (https://www.arabidopsis.org/; accessed on 15 October 2024) and used as queries to perform BLASTP searches against the *P. euphratica* protein database using BLAST (v2.13.0) with an E-value threshold of 1 × 10^−5^. Candidate sequences with sequence identity ≥ 50% and alignment coverage ≥ 50% were retained. Second, the hidden Markov model (HMM) profile of the conserved PLATZ domain (PF04640) was downloaded from the Pfam database (http://pfam.xfam.org/; accessed on 19 October 2024), and HMMER (v3.3.2) was used to search the *P. euphratica* proteome with an E-value cutoff of 1 × 10^−5^. Candidate proteins identified by both methods were combined, and redundant or incomplete sequences were manually removed to obtain the final set of PePLATZ proteins. Afterwards, candidates were validated by using the Conserved Domain Database (http://www.ncbi.nlm.nih.gov/Structure/cdd/wrpsb.cgi; accessed on 19 October 2024) and the SMART online tool (http://smart.embl-heidelberg.de/; accessed on 19 October 2024). This step confirmed the presence and integrity of the PLATZ domain. Sequences lacking the canonical or complete PLATZ domain were excluded. Ultimately, 19 *PePLATZ* genes were identified. The gene positioning visualisation functionality of TBtools software (v2.119) was then applied to show chromosome locations. The physicochemical properties of the *PePLATZ* gene family members were then analysed. These included amino acid number, protein molecular weight, theoretical isoelectric point and hydrophobicity index. The ExPASy website (https://web.expasy.org/protparam/; accessed on 19 October 2024) was used for these analyses. The subcellular localisation of PePLATZ proteins was predicted with the WOLF PSORT website (https://www.genscript.com/).

### 4.2. Phylogenetic and Synteny Analyses

The amino acid sequence clustering and phylogenetic analyses of PLATZ proteins from *P. euphratica*, *P. pruinosa*, *S. sinopurpurea* and *A. thaliana* [58] were conducted by using Clustal W with default settings in MEGA7. A phylogenetic tree was then constructed via the neighbour-joining method with 1000 bootstrap replicates in MEGA7 [59] and visualised by using ITOL (https://itol.embl.de/; accessed on 1 December 2024). Homologous *PLATZ* gene pairs between *P. euphratica* and four reference species, namely, *P. pruinosa*, *P. deltoides*, *S. sinopurpurea* and *A. thaliana*, were identified by using BLASTP. Subsequently, interspecies collinearity was assessed by applying MCScanX after manually removing redundant chromosomes from the CTL file, and the resulting syntenic relationships were visualised by employing TBtools software (v2.119).

### 4.3. Gene Structure, Sequence Motif and Conserved Domain Analyses

This study used the GFF3 annotation file of *P. euphratica* as the primary data source and TBtools software (v2.119). software for a systematic analysis of its gene characteristics. Conserved domain prediction identified key protein domains in the *P. euphratica* genome, whereas gene structure visualisation tools revealed intron and exon genomic distribution patterns. This approach provides a visual framework for investigating the gene function and evolutionary features of *P. euphratica* proteins. Conserved motifs in all PePLATZ protein sequences were predicted using MEME (http://meme-suite.org/tools/meme; accessed on 20 October 2024), with the motif number set to 10 and other parameters at default values. The results were visualised with TBtools software (v2.119).

### 4.4. Promoter Cis-Regulatory Element Analysis

By using the genome annotation and whole-genome sequence of *P. euphratica*, the 2000 bp region upstream of each *PePLATZ* coding sequence was retrieved as the promoter region with TBtools [60]. These promoter sequences were then submitted to the PlantCare database (http://bioinformatics.psb.ugent.be/webtools/plantcare/html/; accessed on 23 October 2024) to predict *cis*-regulatory elements [61]. The PlantCare results were manually curated, simplified and visualised for *cis*-element distribution within promoter regions by utilising TBtools software (v2.119).

### 4.5. Evolutionary Rate and Protein Structure Analysis

The Simple Ka:Ks Calculator (NG) in TBtools software (v2.119) was used to calculate Ka and Ks values for homologous gene pairs within *P. euphratica* to investigate evolutionary constraints on PLATZ genes in poplar. The Ka:Ks values were analysed to determine the selective evolution model of *P. euphratica* [62]. The divergence time (T) of predicted homologous gene pairs was estimated by using the equation T = Ks/2r MYA. For dicotyledonous plants, Ks is the synonymous substitution rate per site, and r is a constant with a value of 1.5 × 10^−8^ [63]. The three-dimensional structure prediction of PePLATZ proteins was conducted with the Normal Model of Phyre2 Server (http://www.sbg.bio.ic.ac.uk/phyre2; accessed on 19 November 2024) [64]. Modelled structures with a confidence value of >85% were then selected.

### 4.6. Analysis of PePLATZ Transcriptome Data

The transcriptome data used in this study were obtained from the authors’ previously published research. *P. euphratica* seedlings were subjected to drought stress through treatment with 25% PEG 6000 solution. Leaf and root tissues were collected at 0, 4 and 12 h after treatment, ands then immediately frozen in liquid nitrogen. RNA sequencing was performed on an Ion Proton platform at the Beijing Genomics Institute (Shenzhen, China) [61]. Gene expression levels were quantified by using the reads per kilobase of transcript per million mapped reads method. The RNA-seq dataset has been deposited in the NCBI Sequence Read Archive under the accession number PRJNA580347 (https://www.ncbi.nlm.nih.gov/bioproject/PRJNA580347/, accessed on 11 September 2024) [61]. Transcriptomic data were visualised by employing TBtools (v2.119), with data normalised and log_2_ transformed (base = 2.0, log = 1.0) prior to analysis.

### 4.7. qRT-PCR Analysis of PePLATZs Under Drought Stress Conditions

One-year-old *P. euphratica* seedlings were used for drought stress assays. Plants were maintained in a controlled growth chamber at 30 °C under a 16 h light/8 h dark photoperiod and 75% relative humidity. Drought stress was induced through irrigation with 20% (*w*/*v*) PEG-6000 solution, whereas control plants received normal watering. Leaf and root tissues were collected at 0, 12 and 24 h after treatment; immediately frozen in liquid nitrogen; and stored at −80 °C until RNA extraction. Total RNA was extracted by using the MiniBEST Plant RNA Extraction Kit (Takara Bio, Otsu, Japan), and first-strand cDNA was synthesised by employing the Goldenstar^TM^ RT6 cDNA Synthesis Kit Ver. 2 (TSINGKE Biotechnology, Beijing, China). qRT-PCR was performed with ChamQ Universal SYBR qPCR Master Mix (Vazyme, Nanjing, China) on a CFX96 Touch Real-Time PCR Detection System (Bio-Rad, USA). Gene-specific primers for *PePLATZ* genes and the reference gene *PeActin* were designed by using NCBI Primer-BLAST (provided in Supplementary Table S3), and their amplification efficiency (90–110%) was validated through standard curve analysis. *PeActin* was used as an internal reference gene due to its stable expression under drought stress conditions in *P. euphratica* [65,66]. Each qRT-PCR reaction was conducted in three technical replicates, and mean values were calculated for subsequent analyses. Relative transcript levels were determined by employing the 2^−ΔΔCt^ method, with expression data presented as mean ± standard deviation. Statistical analyses were performed with GraphPad Prism 8 (GraphPad Software, San Diego, USA) [67].

### 4.8. Protein Interaction Network and Subcellular Localisation Analysis

PPI network analysis was conducted by using the STRING database (https://cn.string-db.org/; accessed on 29 November 2024) [67]. For the determination of the subcellular localisation of PePLATZ8, its coding sequence (excluding the stop codon) was amplified and inserted into the pCAMBIA1300 Super:GFP expression vector, resulting in the fusion construct pCAMBIA1300 Super:PePLATZ8–GFP, in which PePLATZ8 was fused in-frame upstream of the GFP reporter gene. The empty Super1300–GFP vector served as the control. Recombinant and control constructs were independently introduced into Agrobacterium tumefaciens strain GV3101, which was subsequently infiltrated into Nicotiana benthamiana leaves. Following infiltration, the plants were incubated in the dark for 48 h, after which transient GFP expression was observed by using an LSM710 confocal laser scanning microscope (Carl Zeiss 980, Oberkochen, Germany). The primer sequences used for construct generation are provided in Supplementary Table S3.

### 4.9. Transcriptional Activity Assay

Gene-specific primers for PePLATZ8 were designed by using Primer Premier v.6 software. The coding sequence was cloned into the pGBKT7 (BD) vector via homologous recombination. For the transformation, the recombinant plasmid, the empty pGBKT7 vector (negative control) and the pGBKT7–VP16 plasmid (positive control) were each separately mixed with the pGADT7 plasmid. To each plasmid mixture, 5 μL of carrier DNA, 100 μL of 10× LiAc and 400 μL of 50% PEG-3350 were added, and the mixture was mixed gently. Next, the complete mixtures were cotransformed into Y2HGold competent cells. After all components were combined, the cells were spread evenly onto SD/-Trp/-Leu dropout agar plates. The plates were incubated at 30 °C for three days [68]. Single colonies were selected and inoculated into 3 mL of SD/-Trp/-Leu liquid medium. The samples were incubated at 30 °C with shaking at 220 rpm for 16–20 h. Optical density was measured, and the culture was normalised. A total of 2.5 μL of the suspension was spotted onto SD/-Trp/-Leu and SD/-Trp/-His/-Leu+X-α-Gal plates. The plates were incubated inverted at 30 °C for three days. Colony colour and growth were recorded and imaged.

### 4.10. Analysis of PePLATZ8 Promoter Activity

By using the annotated genome of *P. euphratica*, the 2000 bp sequence immediately upstream of the *PePLATZ8* coding region was extracted with TBtools (v2.119) and amplified. The promoter fragment was cloned into the pBI121–GUS reporter vector through homologous recombination. After sequence verification, the recombinant plasmid was introduced into *Agrobacterium*. As described in Method 4.9, *Agrobacterium* suspensions were then infiltrated into the epidermis of *N. benthamiana* leaves. Plants were incubated for 12 h in the dark at room temperature. Subsequently, they were returned to standard growth conditions for 24–36 h before treatment. Leaf discs were exposed to 0.1 mM gibberellic acid, 0.1 mM ABA, or 0.1 mM MeJA for 12 h. For the drought experiment, infiltrations were performed on six-week-old plants subjected to a seven-day water-withholding regime; control infiltrations were performed on well-watered plants. After treatment, histochemical GUS staining was performed, and stained discs were cleared through an ethanol series (75%, 80%, 90% and 95% anhydrous ethanol) before image capture with a flatbed scanner. Primer sequences are provided in Supplementary Table S3.

### 4.11. Acquisition of Transgenic Material

The coding sequence of *PePLATZ8* was amplified and cloned into the Gateway binary vector pGWB2 to generate a C-terminal 5× Myc-tagged overexpression construct driven by the CaMV 35S promoter. The recombinant plasmid was introduced into the *A. tumefaciens* strain GV3101 and used to transform poplar (cv. ‘Nanlin895’) via the leaf disc method. Transgenic shoots were selected on regeneration medium containing the appropriate selectable agent (kanamycin), and putative transformants were rooted and acclimated prior to molecular characterisation. The integration and expression of the *PePLATZ8* transgene were confirmed through analyses at multiple levels. These analyses included (i) genomic PCR to verify T-DNA insertion, (ii) qRT-PCR to assess transcript abundance and (iii) immunoblotting with an anti-Myc antibody to detect the 5× Myc-tagged fusion protein. Nine independent overexpression lines were confirmed by these analyses. Three lines exhibiting the highest *PePLATZ8* transcript and protein levels (as determined by qRT-PCR and immunoblot densitometry) were selected for subsequent drought-tolerance assays. The primer sequences used for cloning and molecular validation are listed in Supplementary Table S3.

### 4.12. Osmotic Stress Analysis of PePLATZ8 Overexpressing Plants

Poplar rooting medium supplemented with 250 mM mannitol was prepared in advance. The apical buds of at least 10 transgenic and WT tissue culture seedlings exhibiting comparable growth statuses were excised and transferred to the mannitol-containing rooting medium. Plant growth was monitored at regular intervals throughout the culture period, and any observable phenotypic differences were documented and photographed.

### 4.13. Analysis of Drought Tolerance in Plants Overexpressing PePLATZ8

Plants were maintained in tissue culture for four weeks. They were then acclimated by removing vessel caps for three days and transplanted into a mixed substrate (nutrient soil:peat soil:vermiculite = 1:1:2). Next, seedlings were grown in an artificial climate chamber (16 h light/8 h dark, 23 °C and 60% relative humidity). Plants were watered regularly, and individuals with the same genotype showing abnormal growth were removed. After approximately 30 days, when plants had reached ~30 cm in height, drought stress was induced by withholding water. Throughout the experiment, transgenic and WT plants were cultivated in parallel, and phenotypic changes were recorded daily. At the conclusion of 18 days of drought treatment, clear phenotypic differences were observed between genotypes. The plants were then rewatered.

The physiological parameters of mature leaves were measured under well-watered and drought conditions lasting seven days. The levels of MDA, SOD, POD, CAT and H_2_O_2_ were quantified by using commercial biochemical assay kits (Solarbio, Beijing, China) in accordance with the manufacturer’s instructions. ROS accumulation was assessed by sampling the third fully expanded leaves of WT and PePLATZ8-OE plants. After drought treatment, leaves were sprayed with distilled water or 100 μM ABA and incubated for 6 h. Samples were then subjected to DAB staining in the dark for 3 h, and brown precipitates in leaf tissues were visualised under a 10× objective lens with a light microscope (Olympus CX21FS1C, Tokyo, Japan). All experiments included at least three independent biological replicates.

### 4.14. Data Processing and Statistical Analysis

Relative gene expression levels were calculated using the 2^−ΔΔCt^ method [69]. All experiments were performed with three biological replicates, and the data are presented as the mean ± standard deviation (SD). Statistical analyses were conducted using Microsoft Excel 2019 and GraphPad Prism 8. Differences among groups were evaluated using one-way analysis of variance (ANOVA) followed by Tukey’s multiple comparison test. Differences were considered statistically significant at *p* < 0.05 (*) and *p* < 0.01 (**).

## 5. Conclusions

This study identified 19 *PePLATZ* genes in the *P. euphratica* genome and divided them into five phylogenetic subclusters. Subsequently, the analysis of gene structure, conserved motifs and protein domains revealed remarkable structural differences between subgroups, indicating functional specialisation. In addition, promoter analysis demonstrated that *PePLATZ* genes harbour *cis*-acting elements related to development, hormone signalling and responses to biotic and abiotic stresses. Moreover, tissue-specific expression patterns suggested differentiated biological roles for individual family members in growth and stress regulation. qRT-PCR, building on these findings, showed that *PePLATZ1*, *PePLATZ6*, *PePLATZ8*, *PePLATZ14* and *PePLATZ19* are responsive to drought. Further analysis revealed that PePLATZ8 localises to the nucleus and lacks intrinsic transcriptional activation activity in yeast. In *poplar*, *PePLATZ8* overexpression enhanced drought tolerance likely by limiting membrane damage and boosting antioxidant enzyme activity. Collectively, these findings provide a comprehensive characterisation of the structural and regulatory features of the *PePLATZ* gene family in *P. euphratica* and identify *PePLATZ8* as a key candidate for improving drought resistance. This work offers valuable insights for the molecular breeding of drought-resilient woody plants.

## Figures and Tables

**Table 1:** Predicted Ka/Ks ratios of *PePLATZ* gene pairs.

Gene 1	Gene 2	Ka	Ks	Ka/Ks	Duplication Date (MYA)
*PePLATZ1*	*PePLATZ6*	0.018289607	0.305553	0.059857473	10.185100
*PePLATZ4*	*PePLATZ15*	0.079327953	0.279809	0.283507714	9.326966
*PePLATZ2*	*PePLATZ15*	0.4362301	-	-	-
*PePLATZ10*	*PePLATZ12*	0.156264029	1.911201	0.081762203	63.706700
*PePLATZ2*	*PePLATZ5*	0.035559443	0.344889	0.103103941	11.496300
*PePLATZ9*	*PePLATZ17*	0.03334383	0.259654	0.128416451	8.655133
*PePLATZ11*	*PePLATZ13*	0.073728707	0.417216	0.176715915	13.907200

## Data Availability

Data will be made available on request.

## Supplementary Materials

The following supporting information can be downloaded at: https://www.mdpi.com/article/10.3390/plants15071065/s1, Figure S1. Analysis of the chromosomal localisation of *PePLATZs*; Figure S2. Protein interaction network of PePLATZ8 protein; Figure S3. Promoter activity analysis of *PePLATZ8*; Table S1. Physicochemical properties of the *PePLATZ* gene family; Table S2. Ka/Ks ratio of homologous gene pairs; Table S3. Primers used in the experiment.
