# Supplementary material for: Genome-Wide Identification of the PLATZ Transcription Factor Family in Populus euphratica Oliv. and Functional Characterisation of PePLATZ8 in Drought Tolerance
Source: Plants (Basel). 2026 Mar 31;15(7):1065. doi: 10.3390/plants15071065 (PMC13074929; doi:10.3390/plants15071065)
Supplement: Supplementary file 1 [file plants-15-01065-s001.zip › Supplementary material1.pdf]

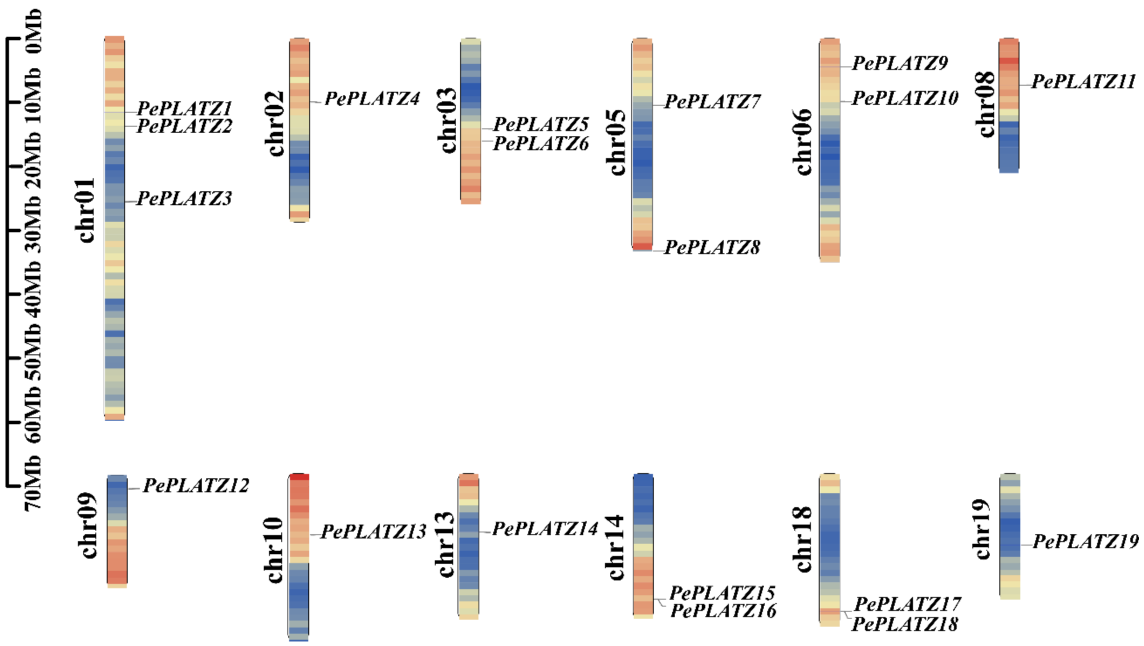

Figure S1. Analysis of the chromosomal localisation of PePLATZs.

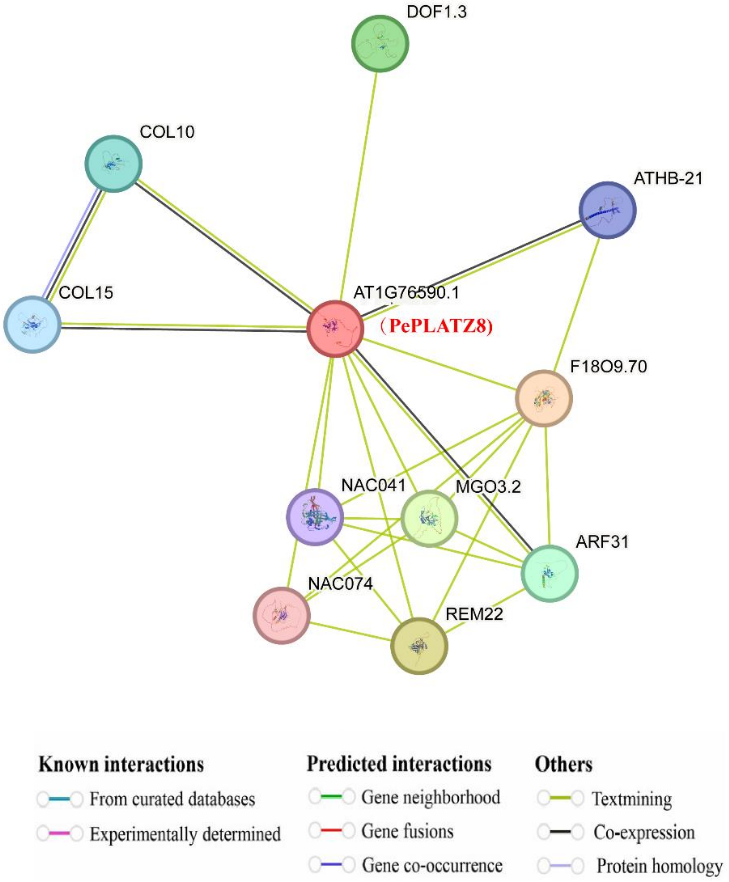

Figure S2. Protein interaction network of PePLATZ8 protein. Proteins are represented as nodes in the network, with each node containing information about related proteins; the color of the lines indicates different data sources.

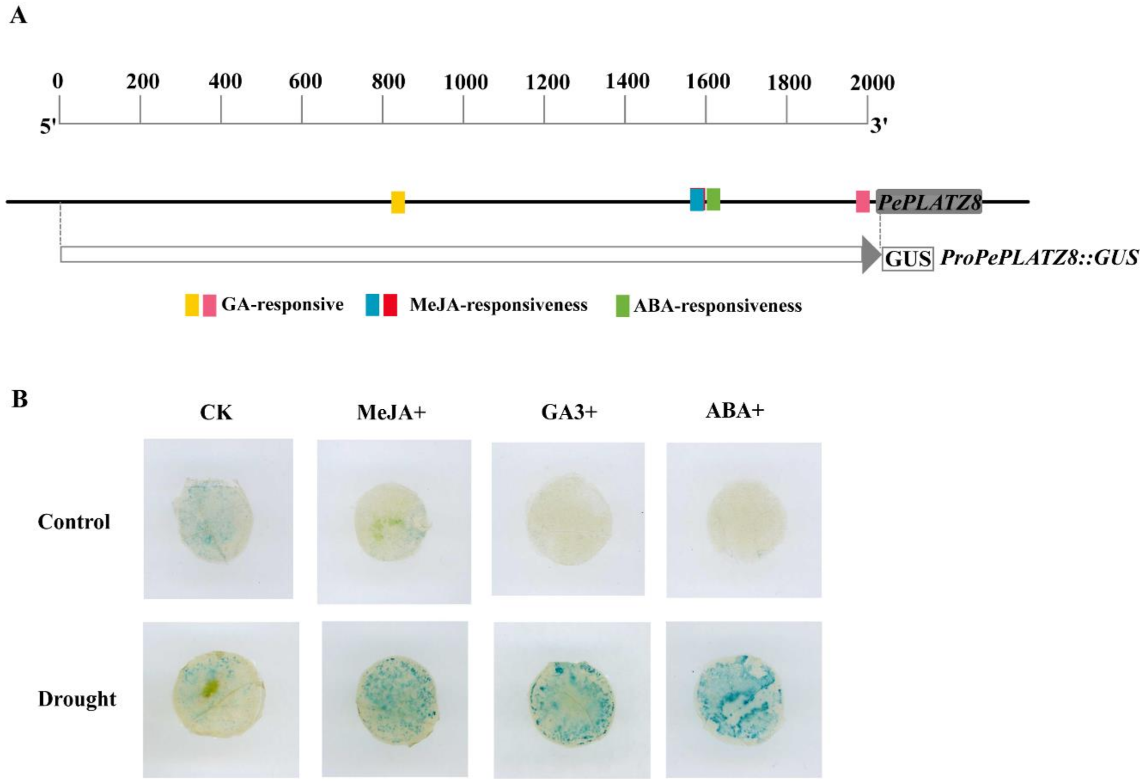

Figure S3. Promoter activity analysis of PePLATZ8. (A) Putative hormone-responsive cis-regulatory elements were predicted within the 2000 bp upstream region of PePLATZ genes; (B) Histochemical analysis of GUS expression driven by the PePLATZ promoters in transgenic tobacco leaves.
