# Supplementary material for: Genome-Wide Identification of the PLATZ Transcription Factor Family in Populus euphratica Oliv. and Functional Characterisation of PePLATZ8 in Drought Tolerance
Source: Plants (Basel). 2026 Mar 31;15(7):1065. doi: 10.3390/plants15071065 (PMC13074929; doi:10.3390/plants15071065)
Supplement: Supplementary file 1 [file plants-15-01065-s001.zip › Supplementary material2.pdf]

Table S1. Physicochemical properties of the *PePLATZ* gene family

Sequence ID	Gene ID	aa	MW （kDa）	pI	Instability Index	Aliphatic Index	GRAVY	subcellular localisation
PeuTF01G01445.1	*PePLATZ1*	173	19.925	9.14	55.92	65.32	-0.629	nucl
PeuTF01G01652.1	*PePLATZ2*	243	27.341	7.53	53.37	75.02	-0.319	nucl
PeuTF01G02130.1	*PePLATZ3*	209	23.840	8.72	67.98	69	-0.57	nucl
PeuTF02G01277.1	*PePLATZ4*	246	27.255	9.34	62.7	87.6	-0.089	chlo
PeuTF03G00643.1	*PePLATZ5*	268	30.364	7.1	59.16	64.7	-0.609	nucl
PeuTF03G00860.1	*PePLATZ6*	223	25.253	9.41	62.81	60.76	-0.681	nucl
PeuTF05G01100.1	*PePLATZ7*	209	24.414	8.91	70.34	59.14	-0.947	nucl
PeuTF05G02404.1	*PePLATZ8*	238	26.757	9.19	53.57	65.55	-0.442	nucl
PeuTF06G00576.1	*PePLATZ9*	243	27.709	8.58	55.89	68.97	-0.479	nucl
PeuTF06G01193.1	*PePLATZ10*	222	25.432	8.69	48.47	100.95	0.3	nucl
PeuTF08G01069.1	*PePLATZ11*	222	25.545	8.78	55.63	70.27	-0.667	nucl
PeuTF09G00048.1	*PePLATZ12*	205	23.327	8.84	61.27	74.63	-0.475	nucl
PeuTF10G01439.1	*PePLATZ13*	317	37.022	8.87	51.65	66.44	-0.545	nucl
PeuTF13G00794.1	*PePLATZ14*	240	27.348	9.2	56.33	67.04	-0.609	nucl
PeuTF14G01368.1	*PePLATZ15*	246	27.550	9.1	57.73	82.85	-0.173	nucl
PeuTF14G01380.1	*PePLATZ16*	246	27.550	9.1	57.73	82.85	-0.173	nucl
PeuTF18G01092.1	*PePLATZ17*	243	27.584	8.77	54.46	72.22	-0.391	nucl
PeuTF18G01097.1	*PePLATZ18*	243	27.584	8.77	54.46	72.22	-0.391	nucl
PeuTF19G00449.1	*PePLATZ19*	240	27.138	9.07	49.88	69.42	-0.575	cyto

Table S2. Ka/Ks ratio of homologous gene pairs

Gene 1	Gene 2	Ka	Ks	Ka/ Ks
PdPLATZ1	*Pe*PLATZ1	0.007392256	0.036873795	0.200474570
PdPLATZ2	*Pe*PLATZ2	0.008823631	0.052081657	0.169419173
PdPLATZ3	*Pe*PLATZ3	0.007398334	0.017217399	0.429701013
PdPLATZ1	*PePLATZ6*	0.037433901	0.312554389	0.119767642
PdPLATZ2	*Pe*PLATZ5	0.039353289	0.326112990	0.120673785
PdPLATZ3	*PePLATZ12*	0.054182160	0.176854074	0.306366478
PdPLATZ2	*PePLATZ15*	0.459251653	3.299097741	0.139205228
PdPLATZ5	*PePLATZ4*	0.019768951	0.065735480	0.300734871
PdPLATZ4	*PePLATZ8*	0.022958634	0.177705348	0.129194951
PdPLATZ5	*PePLATZ15*	0.069851709	0.310365484	0.225062750
PdPLATZ6	*PePLATZ2*	0.029831492	0.324322433	0.091980969
PdPLATZ7	*PePLATZ1*	0.015622175	0.280223373	0.055749007
PdPLATZ6	*PePLATZ5*	0.004766965	0.029531374	0.161420366
PdPLATZ7	*PePLATZ6*	0.005844185	0.020596260	0.283749837
PdPLATZ8	*PePLATZ7*	0.033790688	0.095556470	0.353620017
PdPLATZ9	*PePLATZ8*	0.005438912	0.012572322	0.432609986
PdPLATZ10	*PePLATZ9*	0.007053689	0.100653312	0.070079056
PdPLATZ10	*PePLATZ17*	0.033318804	0.260455627	0.127925069
PdPLATZ11	*PePLATZ11*	0.007689910	0.050560656	0.152092773
PdPLATZ11	*PePLATZ13*	0.069476141	0.380635772	0.182526568
PdPLATZ12	*PePLATZ11*	0.075597417	0.368790072	0.204987668
PdPLATZ12	*PePLATZ13*	0.043520585	0.095279951	0.456765396
PdPLATZ13	*PePLATZ14*	0.024600124	0.042017989	0.585466491
PdPLATZ14	*PePLATZ4*	0.079377386	0.262640216	0.302228602
PdPLATZ14	*PePLATZ15*	0.007094348	0.041968990	0.169037851
PdPLATZ14	*PePLATZ16*	0.007094348	0.041968990	0.169037851
PdPLATZ15	*PePLATZ10*	0.045412942	0.209409925	0.216861462
PdPLATZ15	*PePLATZ12*	0.175951970	1.626077048	0.108206416
PdPLATZ16	*PePLATZ9*	0.029595829	0.271132773	0.109156222
PdPLATZ16	*PePLATZ17*	0.007094348	0.091517085	0.0775193824
PdPLATZ17	*PePLATZ14*	0.062168493	0.218714843	0.2842445099
PdPLATZ17	*PePLATZ19*	0.005344441	0.025976622	0.2057403986
AtPLATZ3	*PePLATZ1*	0.126145230	-	-
AtPLATZ3	*PePLATZ6*	0.174731854	3.097076911	0.056418312
AtPLATZ5	*PePLATZ8*	0.179634974	-	-
AtPLATZ1	*PePLATZ8*	0.149103618	3.417278660	0.043632267
AtPLATZ4	*PePLATZ8*	0.266296633	3.216686056	0.082786019
AtPLATZ8	*PePLATZ10*	0.296382529	-	-

AtPLATZ7	*PePLATZ9*	0.144125334	1.525822082	0.094457497
AtPLATZ6	*PePLATZ11*	0.335581970	2.178470044	0.154044794
AtPLATZ8	*PePLATZ12*	0.200818257	2.019766947	0.099426450
AtPLATZ6	*PePLATZ13*	0.343978199	3.458808205	0.099449920
AtPLATZ7	*PePLATZ17*	0.144715677	1.509690644	0.095857835
AtPLATZ10	*PePLATZ2*	0.423821440	3.097076912	0.136845630
AtPLATZ10	*PePLATZ4*	0.331054950	-	-
AtPLATZ10	*PePLATZ5*	0.423335579	2.397062112	0.176606011
AtPLATZ10	*PePLATZ15*	0.298777198	-	-
AtPLATZ11	*PePLATZ1*	0.171745216	1.376955227	0.124728250
AtPLATZ11	*PePLATZ6*	0.171944298	1.518243113	0.113252151
AtPLATZ12	*PePLATZ1*	0.283080331	1.285526755	0.220205709
AtPLATZ12	*PePLATZ6*	0.290084674	1.9416462629	0.149401402
PpPLATZ1	*PePLATZ1*	0.002452987	0.00909102	0.269825228
PpPLATZ2	*PePLATZ2*	0	0.025453132	0
PpPLATZ3	*PePLATZ3*	0.010413218	0.029024178	0.358777373
PpPLATZ1	*PePLATZ6*	0.031351503	0.346023745	0.090605063
PpPLATZ2	*PePLATZ5*	0.035570541	0.354890079	0.100229741
PpPLATZ3	*PePLATZ12*	0.051903249	0.249975547	0.207633304
PpPLATZ2	*PePLATZ15*	0.4362301	-	-
PpPLATZ14	*PePLATZ11*	0.073373404	0.406874573	0.180334208
PpPLATZ14	*PePLATZ13*	0.011588835	0.021430029	0.540775513
PpPLATZ16	*PePLATZ14*	0.008904824	0.026232182	0.339461814
PpPLATZ17	*PePLATZ4*	0.077337656	0.271795921	0.284543109
PpPLATZ17	*PePLATZ15*	0.001765745	0.023764364	0.074302235
PpPLATZ17	*PePLATZ16*	0.001765745	0.023764364	0.074302235
PpPLATZ19	*PePLATZ19*	0.001777779	0.025948534	0.068511717
PpPLATZ5	*PePLATZ4*	0.014352359	0.023179054	0.619195207
PpPLATZ4	*PePLATZ8*	0.040902328	0.238840298	0.171253881
PpPLATZ5	*PePLATZ15*	0.07436427	0.28483957	0.261074224
PpPLATZ6	*PePLATZ2*	0.035559443	0.334535357	0.106295022
PpPLATZ7	*PePLATZ1*	0.018273691	0.292820423	0.062405792
PpPLATZ6	*PePLATZ5*	0	0.005813983	0
PpPLATZ7	*PePLATZ6*	0	0.006749202	0
PpPLATZ8	*PePLATZ7*	0	0	-
PpPLATZ9	*PePLATZ8*	0.003618825	0	-
PpPLATZ10	*PePLATZ10*	0	0.020237315	0
PpPLATZ10	*PePLATZ12*	0.167527059	1.428985627	0.117234951
PpPLATZ12	*PePLATZ11*	0.001915098	0.02847317	0.067259753
PpPLATZ12	*PePLATZ13*	0.07159941	0.468942057	0.15268285
PpPLATZ13	*PePLATZ3*	0.058629948	0.181831289	0.322441469

PpPLATZ13	*PePLATZ10*	0.174021591	1.792140011	0.097102676
PpPLATZ13	*PePLATZ12*	0.008371207	0.03812765	0.219557369

Table S3. Primers used in the experiment

**Primer name**	**Primer sequence (5'→3')**
proPePLATZ8-F	CTTCCTTCCTGTTTGCCT
proPePLATZ8-R	ATGGTGGACCCATGTCGTGG
proPePLATZ8-F	ATGACCATGATTACGCCCTTCCTTCCTGTTTGCCT
proPePLATZ8-R	ACTGACCACCCGGGGATCCGACTTTGCTATTACTATTAG
T7	TAATACGACTCACTATAGGG
3-BD	TAAGAGTCACTTTAAAATTTGTATAC
BD-PLATZ8-F	tggccatggaggccgaattcATGGTGAGTTCATTTGGG
BD-PLATZ8-R	tcgacggatccccggTTAAAATGGGGCACGATGAGG
Super1300-PLATZ8-F	cggggtcgacatttaaatATGGTGAGTTCATTTGGG
Super1300-PLATZ8-R	ccatggtaccggatccAAATGGGGCACGATGAGG
PLATZ8-F	ATGGTGAGTTCATTTGGGCAA
PLATZ8-R	TTAAAATGGGGCACGATGAGG
topo-PLATZ8-F	CGGCCGCCCCCTTCACCATGGTGAGTTCATTTGGGC
topo-PLATZ8-R	TCGGCGCGCCCACCCTTAAATGGGGCACGATGAGG
PePLATZ1-F	TCTTTGTTCAATGCAAGCAGCA
PePLATZ1-R	GGTCCTTGTGGTAGGCAAGA
PePLATZ6-F	ATTCCTTCCGCTTCTGCTCT
PePLATZ6-R	CTTCGTCTTCATGTACCGCC
PePLATZ8-F	GAGGGATGAAGCAGGGTGAC
PePLATZ8-R	TCTGACCCACCAAAGAAGTGA
PePLATZ14-F	CATGGCTGCATACACTTCTCC
PePLATZ14-R	CCGTTCATGCAATCCAAGCAA
PePLATZ19-F	GAAAGGCATTGGCAACGGAG
PePLATZ19-R	TGTGGAACTCCCTTCCTCCT
PeActin-F	GTCCTCTTCCAGCCATCTC
PeActin-R	TTCGGTCAGCAATACCAGG
